# 2-Phen­oxy-1,2,4-triazolo[1,5-*a*]quinazol­in-5(4*H*)-one

**DOI:** 10.1107/S1600536812021782

**Published:** 2012-05-19

**Authors:** Rashad Al-Salahi, Lolak Nabih, Mohamed Al-Omar, Seik Weng Ng

**Affiliations:** a Department of Pharmaceutical Chemistry, College of Pharmacy, King Saud University, Riyadh 11451, Saudi Arabia; b Department of Chemistry, Institute of Pharmacy, University of Hamburg, Bundesstrasse 45, 20146 Hamburg, Germany; c Department of Chemistry, University of Malaya, 50603 Kuala Lumpur, Malaysia; d Chemistry Department, Faculty of Science, King Abdulaziz University, PO Box 80203 Jeddah, Saudi Arabia

## Abstract

The triazoloquinazole ring system in the title compound, C_15_H_10_N_4_O_2_ is approximately planar (r.m.s. deviation = 0.035 Å). The phenyl ring of the phen­oxy substitutent is aligned at 59.3 (1)° with respect to this ring system. In the crystal, two mol­ecules are linked about a center of inversion by a pair of N—H⋯O hydrogen bonds, generating a dimer.

## Related literature

The synthesis was based on theat of a similar compound; see: Al-Salahi & Geffken (2011 ▶).
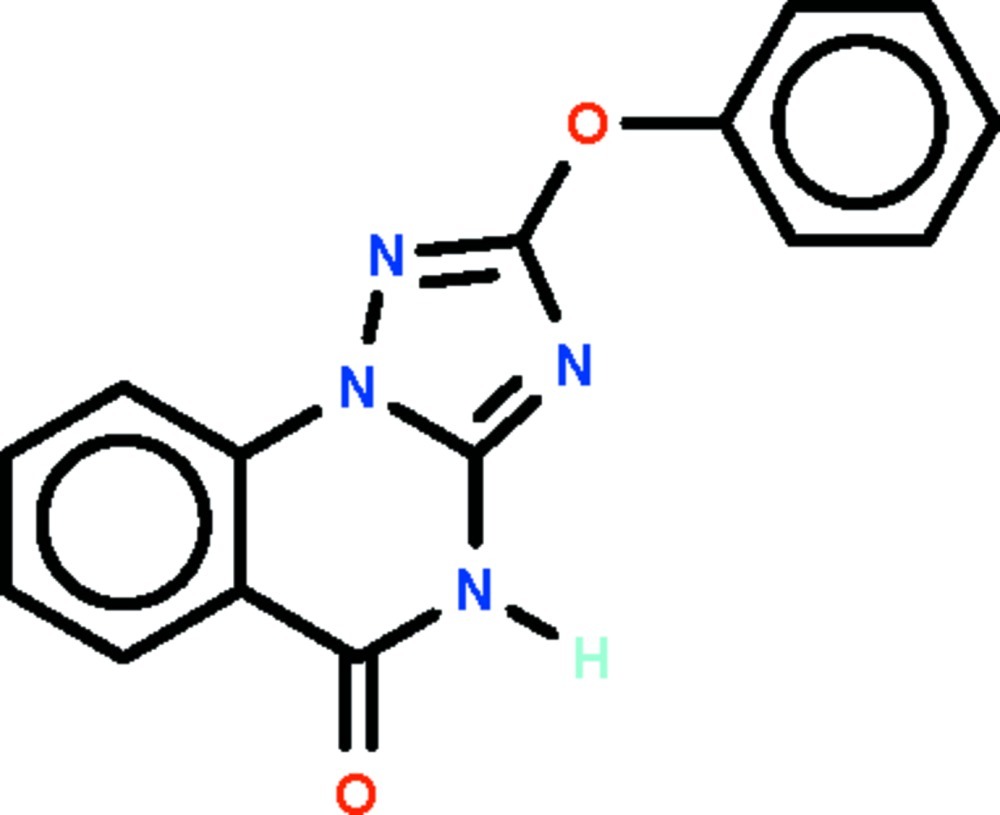


## Experimental

### 

#### Crystal data

C_15_H_10_N_4_O_2_
*M* *_r_* = 278.27
Triclinic, 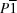

*a* = 5.6985 (2) Å
*b* = 8.4328 (4) Å
*c* = 13.4322 (7) Å
α = 74.087 (4)°
β = 86.623 (4)°
γ = 89.284 (4)°
*V* = 619.66 (5) Å^3^
*Z* = 2
Cu *K*α radiation
μ = 0.86 mm^−1^
*T* = 294 K
0.30 × 0.30 × 0.10 mm

#### Data collection

Agilent SuperNova Dual diffractometer with an Atlas detector
Absorption correction: multi-scan (*CrysAlis PRO*; Agilent, 2012 ▶) *T*_min_ = 0.783, *T*_max_ = 0.919
10219 measured reflections
2570 independent reflections
2408 reflections with *I* > 2σ(*I*)
*R*_int_ = 0.021

#### Refinement

*R*[*F* ^2^ > 2σ(*F* ^2^)] = 0.035
*wR*(*F* ^2^) = 0.103
*S* = 1.03
2570 reflections
194 parameters
1 restraint
H atoms treated by a mixture of independent and constrained refinement
Δρ_max_ = 0.17 e Å^−3^
Δρ_min_ = −0.17 e Å^−3^

### 

Data collection: *CrysAlis PRO* (Agilent, 2012 ▶); cell refinement: *CrysAlis PRO*; data reduction: *CrysAlis PRO*; program(s) used to solve structure: *SHELXS97* (Sheldrick, 2008 ▶); program(s) used to refine structure: *SHELXL97* (Sheldrick, 2008 ▶); molecular graphics: *X-SEED* (Barbour, 2001 ▶); software used to prepare material for publication: *publCIF* (Westrip, 2010 ▶).

## Supplementary Material

Crystal structure: contains datablock(s) global, I. DOI: 10.1107/S1600536812021782/bt5917sup1.cif

Structure factors: contains datablock(s) I. DOI: 10.1107/S1600536812021782/bt5917Isup2.hkl

Supplementary material file. DOI: 10.1107/S1600536812021782/bt5917Isup3.cml

Additional supplementary materials:  crystallographic information; 3D view; checkCIF report

## Figures and Tables

**Table 1:** Hydrogen-bond geometry (Å, °)

*D*—H⋯*A*	*D*—H	H⋯*A*	*D*⋯*A*	*D*—H⋯*A*
N1—H1⋯O1^i^	0.88 (1)	1.90 (1)	2.775 (1)	174 (1)

Symmetry code: (i) 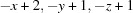.

**Fig. 1.**
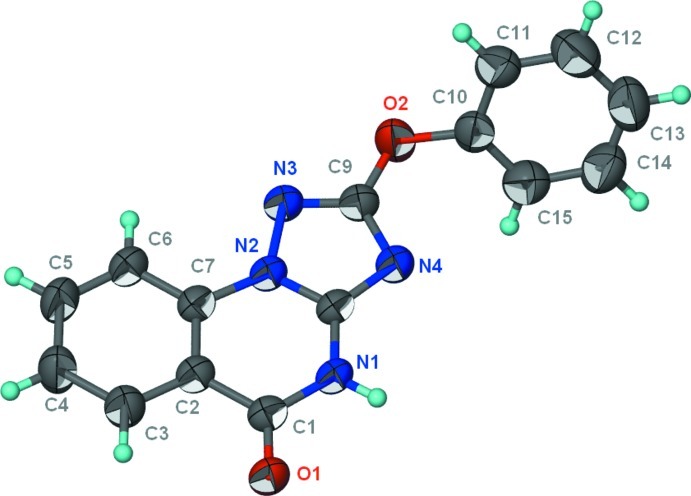
Anitropic displacement ellipsoid plot (Barbour, 2001) of C15H10N4O2 at the 70% probability level; hydrogen atoms are drawn as spheres of arbitrary radius.

## supplementary crystallographic information

### Comment

The procedure for the synthesis of 2-(methylsulfanyl)-[1,2,4]triazolo[1,5-*a*]quinazolin-5-one uses dimethyl *N*-cyanodithioimidocarbonate as one of the reactants (Al-Salahi & Geffken, 2011). The title phenoxy-substituted analog (Scheme I) is obtained with diphenyl *N*-cyanodithioimidocarbonate instead. The triazoloquinazole fused-ring system of C_15_H_10_N_4_O_2_ is planar. The phenyl ring of the phenoxy substitutent is aligned at 59.3 (1) ° with respect to this ring system. Two molecules are linked about a center of inversion by N–H···O hydrogen bonds to generate a dimer (Table 1).

### Experimental

Under ice-cold conditions, 2-hydrazinobenzoic acid (10 mmol, 1.52 g) was added to a solution of diphenyl *N*-cyanodithioimidocarbonate (10 mmol, 2.38 g) in ethanol (20 ml). Triethylamine (30 mmol, 3.03 g) was added. The reaction mixture was stirred overnight at room temperature. Concentrated hydrochloric acid was added; the acidified mixture for heated for an hour. The mixture was poured into ice water; the solid that formed was collected and recrystallized from ethanol to give colorless crystals of 2-phenoxy-[1,2,4]triazolo[1,5-*a*]quinazolin-5-one. The procedure was based on that reported for 2-(methylsulfanyl)-[1,2,4]triazolo[1,5-*a*]quinazolin-5-one (Al-Salahi & Geffken, 2011).

### Refinement

All H-atom were located in a difference Fourier map. Carbon-bound H-atoms were placed in calculated positions [C–H 0.93 Å, *U*_iso_(H) 1.2*U*_eq_(C)] and were included in the refinement in the riding model approximation.

The amino H-atom was refined isotropically with a distance restraint of N–H 0.88±0.01 Å.

### Crystal data

**Table:**

C_15_H_10_N_4_O_2_	*Z* = 2
*M**_r_* = 278.27	*F*(000) = 288
Triclinic, *P*1	*D*_x_ = 1.491 Mg m^−^^3^
Hall symbol: -P 1	Cu *K*α radiation, λ = 1.54184 Å
*a* = 5.6985 (2) Å	Cell parameters from 6342 reflections
*b* = 8.4328 (4) Å	θ = 5.5–76.8°
*c* = 13.4322 (7) Å	µ = 0.86 mm^−^^1^
α = 74.087 (4)°	*T* = 294 K
β = 86.623 (4)°	Prism, colorless
γ = 89.284 (4)°	0.30 × 0.30 × 0.10 mm
*V* = 619.66 (5) Å^3^	

### Data collection

**Table:**

Agilent SuperNova Dual diffractometer with an Atlas detector	2570 independent reflections
Radiation source: SuperNova (Cu) X-ray Source	2408 reflections with *I* > 2σ(*I*)
Mirror monochromator	*R*_int_ = 0.021
Detector resolution: 10.4041 pixels mm^-1^	θ_max_ = 77.0°, θ_min_ = 5.5°
ω scan	*h* = −7→7
Absorption correction: multi-scan (*CrysAlis PRO*; Agilent, 2012)	*k* = −10→10
*T*_min_ = 0.783, *T*_max_ = 0.919	*l* = −16→16
10219 measured reflections	

### Refinement

**Table:**

Refinement on *F*^2^	Primary atom site location: structure-invariant direct methods
Least-squares matrix: full	Secondary atom site location: difference Fourier map
*R*[*F*^2^ > 2σ(*F*^2^)] = 0.035	Hydrogen site location: inferred from neighbouring sites
*wR*(*F*^2^) = 0.103	H atoms treated by a mixture of independent and constrained refinement
*S* = 1.03	*w* = 1/[σ^2^(*F*_o_^2^) + (0.0596*P*)^2^ + 0.101*P*] where *P* = (*F*_o_^2^ + 2*F*_c_^2^)/3
2570 reflections	(Δ/σ)_max_ = 0.001
194 parameters	Δρ_max_ = 0.17 e Å^−^^3^
1 restraint	Δρ_min_ = −0.17 e Å^−^^3^

### Fractional atomic coordinates and isotropic or equivalent isotropic displacement parameters (Å^2^)

**Table:**

	*x*	*y*	*z*	*U*_iso_*/*U*_eq_	
O1	0.99321 (13)	0.65595 (10)	0.38673 (6)	0.0434 (2)	
O2	0.05541 (15)	0.63656 (11)	0.73718 (7)	0.0575 (3)	
N1	0.72791 (14)	0.61117 (11)	0.52356 (7)	0.0368 (2)	
H1	0.815 (2)	0.5287 (14)	0.5565 (10)	0.052 (4)*	
N2	0.39197 (14)	0.77669 (10)	0.51740 (7)	0.0357 (2)	
N3	0.19077 (15)	0.78851 (11)	0.57764 (7)	0.0407 (2)	
N4	0.42432 (15)	0.57551 (11)	0.66105 (7)	0.0392 (2)	
C1	0.80686 (17)	0.69434 (12)	0.42497 (8)	0.0357 (2)	
C2	0.65586 (18)	0.82833 (13)	0.36907 (8)	0.0371 (2)	
C3	0.7175 (2)	0.91440 (15)	0.26696 (9)	0.0478 (3)	
H3A	0.8541	0.8874	0.2340	0.057*	
C4	0.5757 (2)	1.03986 (17)	0.21472 (10)	0.0559 (3)	
H4	0.6164	1.0970	0.1463	0.067*	
C5	0.3716 (2)	1.08128 (15)	0.26419 (10)	0.0509 (3)	
H5	0.2790	1.1674	0.2285	0.061*	
C6	0.30459 (19)	0.99747 (14)	0.36443 (9)	0.0423 (3)	
H6	0.1673	1.0250	0.3967	0.051*	
C7	0.44778 (18)	0.86996 (13)	0.41660 (8)	0.0354 (2)	
C8	0.52273 (17)	0.64985 (12)	0.56919 (8)	0.0342 (2)	
C9	0.22415 (18)	0.66579 (14)	0.66014 (8)	0.0400 (2)	
C10	0.0660 (2)	0.49527 (15)	0.82080 (9)	0.0453 (3)	
C11	−0.1218 (2)	0.38863 (17)	0.83833 (10)	0.0524 (3)	
H11	−0.2437	0.4075	0.7934	0.063*	
C12	−0.1261 (3)	0.25285 (19)	0.92387 (11)	0.0621 (4)	
H12	−0.2514	0.1790	0.9366	0.075*	
C13	0.0546 (3)	0.22606 (19)	0.99069 (11)	0.0664 (4)	
H13	0.0512	0.1346	1.0482	0.080*	
C14	0.2391 (3)	0.3353 (2)	0.97164 (11)	0.0663 (4)	
H14	0.3603	0.3175	1.0169	0.080*	
C15	0.2473 (2)	0.47089 (19)	0.88648 (11)	0.0567 (3)	
H15	0.3729	0.5445	0.8736	0.068*	

### Atomic displacement parameters (Å^2^)

**Table:**

	*U*^11^	*U*^22^	*U*^33^	*U*^12^	*U*^13^	*U*^23^
O1	0.0337 (4)	0.0480 (4)	0.0435 (4)	0.0121 (3)	0.0022 (3)	−0.0057 (3)
O2	0.0464 (5)	0.0600 (5)	0.0506 (5)	0.0199 (4)	0.0148 (4)	0.0065 (4)
N1	0.0287 (4)	0.0391 (5)	0.0391 (5)	0.0091 (3)	−0.0027 (3)	−0.0053 (4)
N2	0.0288 (4)	0.0379 (5)	0.0377 (5)	0.0075 (3)	−0.0014 (3)	−0.0064 (4)
N3	0.0319 (4)	0.0454 (5)	0.0410 (5)	0.0097 (4)	0.0023 (4)	−0.0066 (4)
N4	0.0330 (4)	0.0414 (5)	0.0391 (5)	0.0073 (3)	0.0000 (4)	−0.0048 (4)
C1	0.0299 (5)	0.0376 (5)	0.0387 (5)	0.0045 (4)	−0.0023 (4)	−0.0091 (4)
C2	0.0323 (5)	0.0376 (5)	0.0395 (5)	0.0055 (4)	−0.0028 (4)	−0.0076 (4)
C3	0.0434 (6)	0.0502 (6)	0.0433 (6)	0.0114 (5)	0.0036 (5)	−0.0038 (5)
C4	0.0558 (7)	0.0581 (7)	0.0420 (6)	0.0153 (6)	0.0037 (5)	0.0044 (5)
C5	0.0482 (7)	0.0486 (6)	0.0481 (7)	0.0159 (5)	−0.0059 (5)	0.0000 (5)
C6	0.0356 (5)	0.0414 (6)	0.0461 (6)	0.0098 (4)	−0.0036 (4)	−0.0058 (5)
C7	0.0313 (5)	0.0357 (5)	0.0379 (5)	0.0035 (4)	−0.0036 (4)	−0.0077 (4)
C8	0.0280 (5)	0.0360 (5)	0.0377 (5)	0.0052 (4)	−0.0042 (4)	−0.0085 (4)
C9	0.0322 (5)	0.0442 (6)	0.0401 (5)	0.0066 (4)	0.0025 (4)	−0.0069 (4)
C10	0.0416 (6)	0.0515 (6)	0.0373 (6)	0.0114 (5)	0.0062 (4)	−0.0053 (5)
C11	0.0428 (6)	0.0679 (8)	0.0431 (6)	0.0044 (5)	0.0020 (5)	−0.0104 (6)
C12	0.0593 (8)	0.0625 (8)	0.0578 (8)	−0.0047 (6)	0.0123 (6)	−0.0082 (6)
C13	0.0724 (9)	0.0666 (9)	0.0466 (7)	0.0142 (7)	0.0078 (6)	0.0043 (6)
C14	0.0580 (8)	0.0876 (11)	0.0464 (7)	0.0160 (7)	−0.0100 (6)	−0.0061 (7)
C15	0.0468 (7)	0.0675 (8)	0.0525 (7)	0.0017 (6)	−0.0024 (5)	−0.0112 (6)

### Geometric parameters (Å, º)

**Table:**

O1—C1	1.2307 (12)	C4—C5	1.3938 (17)
O2—C9	1.3435 (13)	C4—H4	0.9300
O2—C10	1.3990 (14)	C5—C6	1.3721 (17)
N1—C8	1.3656 (13)	C5—H5	0.9300
N1—C1	1.3699 (13)	C6—C7	1.3947 (14)
N1—H1	0.878 (9)	C6—H6	0.9300
N2—C8	1.3477 (12)	C10—C15	1.3753 (18)
N2—N3	1.3824 (12)	C10—C11	1.3738 (18)
N2—C7	1.3874 (14)	C11—C12	1.3813 (19)
N3—C9	1.3139 (14)	C11—H11	0.9300
N4—C8	1.3164 (14)	C12—C13	1.382 (2)
N4—C9	1.3622 (13)	C12—H12	0.9300
C1—C2	1.4722 (14)	C13—C14	1.372 (2)
C2—C3	1.3910 (16)	C13—H13	0.9300
C2—C7	1.4000 (14)	C14—C15	1.377 (2)
C3—C4	1.3794 (17)	C14—H14	0.9300
C3—H3A	0.9300	C15—H15	0.9300
			
C9—O2—C10	119.95 (9)	N2—C7—C6	122.26 (10)
C8—N1—C1	122.69 (8)	N2—C7—C2	116.34 (9)
C8—N1—H1	120.6 (9)	C6—C7—C2	121.40 (10)
C1—N1—H1	116.7 (9)	N4—C8—N2	111.92 (9)
C8—N2—N3	109.16 (8)	N4—C8—N1	128.29 (9)
C8—N2—C7	123.88 (9)	N2—C8—N1	119.77 (9)
N3—N2—C7	126.79 (8)	N3—C9—O2	117.36 (9)
C9—N3—N2	100.32 (8)	N3—C9—N4	118.04 (9)
C8—N4—C9	100.54 (8)	O2—C9—N4	124.59 (10)
O1—C1—N1	120.70 (9)	C15—C10—C11	121.75 (12)
O1—C1—C2	123.08 (10)	C15—C10—O2	121.40 (12)
N1—C1—C2	116.22 (9)	C11—C10—O2	116.68 (11)
C3—C2—C7	118.94 (10)	C10—C11—C12	118.73 (12)
C3—C2—C1	120.02 (10)	C10—C11—H11	120.6
C7—C2—C1	121.04 (10)	C12—C11—H11	120.6
C4—C3—C2	119.93 (11)	C11—C12—C13	120.38 (14)
C4—C3—H3A	120.0	C11—C12—H12	119.8
C2—C3—H3A	120.0	C13—C12—H12	119.8
C3—C4—C5	120.17 (12)	C14—C13—C12	119.61 (13)
C3—C4—H4	119.9	C14—C13—H13	120.2
C5—C4—H4	119.9	C12—C13—H13	120.2
C6—C5—C4	121.30 (11)	C13—C14—C15	120.89 (13)
C6—C5—H5	119.4	C13—C14—H14	119.6
C4—C5—H5	119.4	C15—C14—H14	119.6
C5—C6—C7	118.26 (11)	C10—C15—C14	118.62 (13)
C5—C6—H6	120.9	C10—C15—H15	120.7
C7—C6—H6	120.9	C14—C15—H15	120.7
			
C8—N2—N3—C9	0.14 (11)	C9—N4—C8—N1	−177.91 (10)
C7—N2—N3—C9	175.59 (10)	N3—N2—C8—N4	−0.50 (12)
C8—N1—C1—O1	179.78 (9)	C7—N2—C8—N4	−176.12 (9)
C8—N1—C1—C2	−0.77 (15)	N3—N2—C8—N1	178.16 (8)
O1—C1—C2—C3	2.27 (17)	C7—N2—C8—N1	2.54 (16)
N1—C1—C2—C3	−177.17 (10)	C1—N1—C8—N4	177.01 (10)
O1—C1—C2—C7	−178.59 (10)	C1—N1—C8—N2	−1.40 (15)
N1—C1—C2—C7	1.98 (15)	N2—N3—C9—O2	−178.60 (10)
C7—C2—C3—C4	0.81 (19)	N2—N3—C9—N4	0.27 (13)
C1—C2—C3—C4	179.98 (12)	C10—O2—C9—N3	171.70 (11)
C2—C3—C4—C5	0.4 (2)	C10—O2—C9—N4	−7.09 (18)
C3—C4—C5—C6	−1.2 (2)	C8—N4—C9—N3	−0.55 (13)
C4—C5—C6—C7	0.7 (2)	C8—N4—C9—O2	178.22 (11)
C8—N2—C7—C6	178.15 (10)	C9—O2—C10—C15	63.17 (17)
N3—N2—C7—C6	3.32 (17)	C9—O2—C10—C11	−121.42 (12)
C8—N2—C7—C2	−1.29 (15)	C15—C10—C11—C12	−0.6 (2)
N3—N2—C7—C2	−176.12 (9)	O2—C10—C11—C12	−175.96 (11)
C5—C6—C7—N2	−178.86 (10)	C10—C11—C12—C13	0.5 (2)
C5—C6—C7—C2	0.55 (17)	C11—C12—C13—C14	0.0 (2)
C3—C2—C7—N2	178.15 (9)	C12—C13—C14—C15	−0.3 (2)
C1—C2—C7—N2	−1.00 (15)	C11—C10—C15—C14	0.2 (2)
C3—C2—C7—C6	−1.29 (17)	O2—C10—C15—C14	175.40 (12)
C1—C2—C7—C6	179.55 (10)	C13—C14—C15—C10	0.2 (2)
C9—N4—C8—N2	0.60 (12)		

### Hydrogen-bond geometry (Å, º)

**Table:**

*D*—H···*A*	*D*—H	H···*A*	*D*···*A*	*D*—H···*A*
N1—H1···O1^i^	0.88 (1)	1.90 (1)	2.775 (1)	174 (1)

Symmetry code: (i) −*x*+2, −*y*+1, −*z*+1.
